# Medical Device Product Innovation Choices in Asia: An Empirical Analysis Based on Product Space

**DOI:** 10.3389/fpubh.2022.871575

**Published:** 2022-04-13

**Authors:** Feng Hu, Liping Qiu, Haiyan Zhou

**Affiliations:** ^1^Global Value Chain Research Center, Zhejiang Gongshang University, Hangzhou, China; ^2^School of Business Administration, Zhejiang Gongshang University, Hangzhou, China; ^3^Institute of Artificial Intelligence and Change Management, Shanghai University of International Business and Economics, Shanghai, China

**Keywords:** medical device products, product space, innovation, potential products, potential opportunity gains

## Abstract

Due to the increasing demand for health care, identifying and evaluating the feasibility of local medical device innovation and production is an important guarantee for the long-term sustainable development of a national health system, especially for Asian countries/regions that are plagued by aging populations. This article analyzes the international trade data of 46 HS 6-digit medical device products exported from 49 countries from 1999 to 2019, and constructs a global medical device product space. Furthermore, the innovation potential and opportunities of potential medical device products in major Asian countries are evaluated by examining the dynamic relationship between the product distance and the acquisition of comparative advantages for medical device products based on an empirical model. The regression results suggest that a close product distance improves the feasibility of developing a new medical device product. The smaller the product distance is, the more likely it is to increase the diversity of the medical device products of a country by maintaining the existing comparative advantages and gaining potential comparative advantages. Furthermore, we follow the conclusions of the empirical model and analyze the product space evolution, and potential product distance and gains of major Asian medical device exporters. These conclusions may help entrepreneurs identify potential development directions and help government policy-makers formulate policies that are in line with national realities.

## Introduction

The outbreak of the COVID-19 pandemic has become a major global health concern, greatly raising awareness of the importance of health (1). However, knowledge about this novel virus is still limited (2), leading to increasingly complex global health challenges. The Sustainable Development Goal 3 to ensure healthy lives and promote well-being for all at all ages requires safe, effective and appropriate medical devices for prevention, early diagnosis, and effective treatment of disease [(3), p. 6]. The importance of health technology, especially medical devices, cannot be overstated.

With the ongoing impact of the COVID-19 pandemic on global markets, supply chain disruptions have led to shortages of critical medical devices worldwide. The sudden surge in demand for some medical devices, such as ventilators, has forced many countries to reduce import controls to address the urgent needs of patients and health care systems. It is generally believed that factors such as global population aging, increased life expectancy, the threat of chronic diseases, and the emergence of new technologies in the market have promoted awareness of the importance of obtaining medical devices. There is a growing demand for medical devices for disease diagnosis, treatment, monitoring, and management. This growing global demand for health increases opportunities for national economic development. However, such opportunities are largely concentrated in high-income countries and have minimal impact on low- and middle-income countries [(4), p. 4].

The development of medical devices has historically been concentrated in high-income countries (5) because it is both expensive and risky due to its high complexity (6). Most low- and middle-income countries do not manufacture medical device products themselves but transfer such products from developed to developing countries through low-cost sales or the donation of existing products (technology diffusion) (7). However, it has been reported that ~40% of medical devices in underresourced areas are unusable (8). This purchasing model has a high risk of technical failure. Therefore, it is necessary to systematically evaluate the feasibility of producing and manufacturing medical devices in a specific environment and improve medical device development through local production.

To this end, this study considers the Asian region. On the one hand, the Asian region contains some of the most dynamic economies in the world, some of which are already at the forefront of global technological development and have great potential for development in the medical device industry. On the other hand, East and Southeast Asia have the largest elderly population in the world (9) and low birth rates in parts of Asia have reduced future labor availability. The current level of medical device development, and national systems and guarantees cannot fully meet the growing health needs of the population. Medical device innovation may become one of the bottlenecks for the long-term sustainable development of Asian health systems.

This study addresses the above issue by introducing a cutting-edge approach involving complexity economics, while also considering product space, which can determine whether it is possible for a country to develop a specific product (10). It has been shown that the success of a new product depends on how close the new product is in the product space to the products that the country has been able to manufacture and export (10–12). In the product space, cross-country heterogeneity is fully considered. The distance between existing products and potential products in each country reflects the differences in production capacity among countries. This distance not only determines the path and extent of industrial transformation, but also implies an important message, that is, the strategy of companies, markets, and countries to determine the direction of innovation.

To support the subsequent evaluation of innovation potential and opportunities of potential medical device products in major Asian countries, we first consider whether the dynamic relationship between the product space and the acquisition of comparative advantages for medical device products is consistent with existing research. We calculate the product space indices for 46 medical device products exported from 49 countries from 1999 to 2019. Regression results confirm that shorter distances in the medical device product space have a positive impact on the acquisition of comparative advantage. Through correlation decomposition, it is found that shorter product distances promote industrial upgrading by gaining comparative advantage and preventing products from losing the advantage. On this basis, we extend the empirical conclusions, identify the evolution patterns of medical device product space in China, Japan, Singapore, Malaysia, and South Korea, and evaluate the options for potential product innovation in the above countries. It may provide guidance for corporate decision-makers and policy-makers in terms of medical device product support and industrial structure adjustment.

The remainder of the article is structured as follows: Section Theoretical Basis and Hypotheses outlines the theoretical background and presents the hypotheses. Section Data Sources and Research Methods describes the variables and regression models. Section Empirical Results presents regression analysis. Section Product Space Analysis of Medical Devices in Asia discusses medical device product innovation choices in Asia. Section Conclusions and Directions for Further Research presents the conclusions, limitations, and directions for further research.

## Theoretical Basis and Hypotheses

### Research on Medical Device Products and Innovations

A medical device is defined as an article, instrument, device or machine used to prevent, diagnose or treat disease, or to detect, measure, restore, correct or modify the structure or function of the body for a health purpose. Its primary mode of action is not based on pharmacological, metabolic or immune processes (13). Medical devices defined in this way are numerous and contain a very diverse set of technologies (14). Innovation is a complex interaction in a technical knowledge base to generate new and nondeterministic processes (15). Reasonable allocation of technical knowledge resources within an organization and timely adjustment of the structure are very important (16). It should be noted that there are multiple definitions of innovation related to medical devices (17). The medical device innovation discussed in this study continues the concept of Schumpeterian innovation, that is, new products start out as inventions and turn into innovations as they are commercialized (18).

As mentioned in the Introduction, the main source of medical device innovation remains limited to high-income countries. As the world's largest medical device market (19), the United States is constrained by limited availability of venture capital and slow commercial rates, etc., resulting in “industry outflow” (20). This also means that many medical device products exported by developing countries may also be manufactured for high-income countries. Compared to innovations from the technological frontier in high-income countries, developing countries are in a relatively resource-scarce environment. This resource-constrained business model and innovation, namely frugal innovation, has received extensive attention from academia (21, 22).

Medical device innovation and its economic evaluation involve many complex factors (14) and stakeholders (20). Stringent regulations make medical device design more complex, and successful medical device innovations often go beyond the functionality of the product itself (23). Kirisits and Redekop (14) suggested that the economic evaluation of medical devices should consider both external factors (regulatory framework, industry structure, short life cycle, and early market diffusion) and device-level factors (design constraints, program integration, repeatability, dynamic efficiency, and resource usage). Medical device product innovation requires a complex assessment of the possibility of industrialization of a new local medical device product. This innovation requires not only the development of technology but also a systematic assessment, including needs, the usage environment and market factors [(4), pp. 18–19], to promote the effective development and use of technology. Therefore, researchers encourage the use of systematic viewpoints to make comprehensive evaluations (22, 24, 25) and pay extensive attention to the medical device background by researching in the elements of medical device innovation.

### Medical Device Product Space

In terms of medical technology, innovation can be divided into the most common evolutionary innovation and the rare revolutionary innovation (26). Evolutionary innovation is the cumulative learning or experience gained through the use of the technology. Gelijns and Rosenberg (27) compared the dynamic interaction view of technological change with the linear model of medical innovation and found that one of the characteristics of medical device innovation is path dependence. The rare revolutionary innovation may come from technology diffusion across disciplines. In other words, medical device innovation is a dynamic and complex process. From idea to invention, to development, and to the commercialization of medical device products, the evolutionary innovations are finally expressed as marketable products.

Product space theory argues that industrial upgrading is not continuous, and its space evolution is formed by technological, organizational and institutional capabilities accumulated at the local level (28). Foreign trade data can be assessed as outcome indicators arising from the impact of a number of market-related parameters. This means that successful innovation will be reflected in the acquisition of comparative advantages by export products. The generation of comparative advantages requires not only the support of technology, but also the support of manpower, material, knowledge, policies and systems. It can be considered that the product space is in line with the suggestion for medical device innovation that the possibility of industrialization of a new local medical device product should be evaluated from a systematic perspective.

Hidalgo and Hausmann (29) investigated the evolution of a country's comparative advantage based on the product space, and found that product distance has an important impact on the evolution of a national or regional industry. Technology-related products have similar requirements for various production factors, production technology, and management experience. Whether a country can realize the transfer of product comparative advantage depends not only on the current capacity endowment and the similarity between the capacity endowment required for the production of new products and that for existing products (similarity of local factors), but also on the government's guidance for industrial capacity endowment (30). The more similar the capacity endowment required to produce a new product is to that accumulated by the existing product, the closer the two products are in the product space. This has been supported in studies of specific industries, such as the biotechnology industry (31), the European nanotechnology industry (32), and the green industry (11). In summary, the successful development of a new product is more likely to occur in areas with an existing product with a similar knowledge (technical) base. Hence, the following hypothesis is proposed:

H1: In the medical device product space, a close distance improves the feasibility of new product development.

If many countries have comparative advantages in two particular products, it indicates that the two products require similar capabilities and can be regarded as related products (10). The deepening of the correlation between products and the derived diversity are the fundamental driving forces which stimulate national industrial upgrading. Specifically, the factor endowment structure determines the industry distribution and development direction. Combined with the characteristics of medical device product innovation, the factor endowment of regional production has two main mechanisms for the evolution of comparative advantage: For products with comparative advantages, accumulating experience and deepening the relationship between products based on existing technologies will enhance the comparative advantage. For products that do not have comparative advantages, the closer they are to products with comparative advantages, the greater the relationship between them, and the greater the opportunity to stimulate product derivative innovations. In summary, related product innovation can be regarded as a recombination of existing capabilities. Continuous recombination not only consolidates the position of advantage products but also expands new and diverse derivative products. Hence, the following hypothesis is proposed:

H2: In the medical device product space, a close product distance helps maintain the existing comparative advantages and promotes product innovation by gaining potential comparative advantages to enhance the diversity of medical device products of a country.

## Data Sources and Research Methods

### Product Space Approach

The specialization matrix organizes the location information of the economic activities in which the country has comparative advantages in the form of a matrix (33), and indicates whether a country has a comparative advantage in product production through revealed comparative advantage (*RCA*). *X*_*cp*_ is the total exports of product *P* from country *C*. The revealed comparative advantage of product *P* of country *C* is expressed as:


$$\begin{array}{l} RCA_{cp}\;=\;\frac{\frac{X_{cp}}{\underset{c}{\sum }X_{cp}}}{\frac{\underset{p}{\sum }X_{cp}}{\underset{c}{\sum }\underset{p}{\sum }X_{cp}}} \end{array}$$

When dealing with the specialization matrix, the product space approach often further defines it as a binary specialization matrix. Hidalgo and Hausmann (29) suggested using *RCA*_*cp*_≥1 as the threshold of national product specialization. The binary specialization matrix *M*_*cp*_ is expressed as follows:


$$M_{cp}\;=\;\{\begin{array}{c} 1\;RCA_{cp}\geq 1\\ 0\;otherwise \end{array}$$

The row sum of *M*_*cp*_ describes the diversity of export products of a country (*Diversity*), which measures how many different types of products a country produces. It is defined as follows:


$$\begin{array}{l} k_{c,0}\;=\;\underset{p}{\sum }M_{cp} \end{array}$$

The column sum of *M*_*cp*_ describes the ubiquity of export products (*Ubiquity*), which measures the number of countries producing a certain product. It is defined as follows:


$$\begin{array}{l} k_{p,0}\;=\;\underset{c}{\sum }M_{cp} \end{array}$$

On the basis of the specialization matrix, Hidalgo et al. (10) defined a *p*×*p* product proximity (*Proximity*) matrix, $\Phi_{pp^{{}^{\prime }}}$, that is, there is a globally defined proximity between every two products to represent the distance between them in a certain period. They suggested that there is a high proximity between two products requiring similar production capacity. $\Phi_{pp^{{}^{\prime }}}$ is measured by the possibility of joint export. The greater the probability that a country exporting product *p* also exports product *p*′, the closer the two products are.


$$\begin{array}{l} \Phi_{pp^{{}^{\prime }}}\;=\;\frac{\underset{c}{\sum }M_{cp}M_{cp^{{}^{\prime }}}}{\max\left(k_{p,0},k_{p^{{}^{\prime }},0}\right)} \end{array}$$

Moreover, this globally defined matrix is widely expressed as a product space network map using a visual network. This expression visually simulates the evolution of a country's production structure and allows easy mapping of the productive capacity of that country. The construction method and information identification will be discussed in detail in Section Product Space Evolution.

The knowledge capacity of each country to produce a product is different. Therefore, it is necessary to measure the distance between the product and the existing production structure of the country. The product proximity $\Phi_{pp^{{}^{\prime }}}$ is fixed on a global scale. The distance matrix (*Distance*) can be defined as the distance of a product being the sum of the proximities of the given product to all other products that are not currently exported at that location:


$$\begin{array}{l} D_{cp}\;=\;\frac{\underset{p^{{}^{\prime }}}{\sum }\left(1\;-\;M_{cp^{{}^{\prime }}}\right)\Phi_{p,p^{{}^{\prime }}}}{\underset{p^{{}^{\prime }}}{\sum }\Phi_{pp^{{}^{\prime }}}} \end{array}$$

Finally, on the basis of the above product space indices, we further analyze the potential development index of the national industry. In other words, if industrial upgrading follows the evolution of comparative advantages, we will examine the potential of products that do not have comparative advantages in the country in the current period.

We define the potential distance *PD*_*cp*_, which is calculated as the dot product of the distance matrix *D*_*cp*_ and the binary specialization matrix *M*_*cp*_, and define the distance of the advantage product as 0, thus clearly showing the proximity of the country's current nonadvantage product to the advantage product.

Second, we define the potential product gain matrix *POG*_*cp*_, which is calculated as the dot product of the opportunity gain matrix *OG*_*cp*_ and the binary specialization matrix *M*_*cp*_. Opportunity gains (*OG*) measure the gains to a country for opening up future diversification opportunities from developing a particular product. The increase in opportunity gains explains the complexity and distance of a product that is not produced in a country or how close the new product is to existing functionality. It is defined by Hausmann et al. [(34), p. 54] as:


$$\begin{array}{l} OG_{cp}\;=\;\sum_{p^{{}^{\prime }}}\frac{\Phi_{p,p^{{}^{\prime }}}}{\underset{p^{{}^{\prime \prime }}}{\sum }\Phi_{p^{{}^{\prime \prime }},p^{{}^{\prime }}}}\left(1-M_{cp^{{}^{\prime }}}\right)PCI_{p^{{}^{\prime }}} \end{array}$$

The product complexity index (*PCI*), i.e., the quantity and complexity of the know-how required to produce a product, is calculated according to Hidalgo and Hausmann (29).

We first construct the matrix ${\tilde{M}}_{p,p^{{}^{\prime }}}^{P}$:


$$\begin{array}{l} {\tilde{M}}_{p,p^{{}^{\prime }}}^{P}\equiv \underset{c}{\sum }\frac{M_{cp}M_{cp^{{}^{\prime }}}}{k_{c,0}k_{p,0}} \end{array}$$

Next, we find the matrix eigenvalue $\vec{q}$ and the corresponding eigenvector $\vec{Q}$, extract the eigenvector ${\vec{Q}}_{l2}$ corresponding to the second largest eigenvalue λ_*l*2_, and obtain the *PCI* calculation formula:


$$\begin{array}{l} PCI_{cp}\;=\;\frac{{\vec{Q}}_{l2}\;-\;average\left(\vec{Q}\right)}{stdev\left(\vec{Q}\right)} \end{array}$$

### Variables and Models

To test the hypotheses to support further analysis, we use the econometric model implemented by Hausmann and Klinger (28) to investigate the product space, which in this case is the dynamic relationship between the product distance and the acquisition of comparative advantages. It is expressed by Equation (1):


(1) $$\begin{array}{r} y_{c,p,t}\;=\;\beta_{0}+\beta_{1}y_{c,p,t\;-\;1}\;+\;\beta_{2}Distance_{c,p,t\;-\;1}\;+\;\beta_{3}X\;+\;\varepsilon \end{array}$$

where the explained variable *y*_*c, p, t*_ is the comparative advantage of product *p* of country *c* in year *t*, which is represented by the binary specialization matrix *M*_*cp, t*_ in year *t*; the explanatory variable *y*_*c, p, t*−1_ is the comparative advantage *M*_*cp, t*−1_ of product *p* of country *c* in the previous period; *Distance*_*c, p, t*−1_ is the distance between product *p* in country *c* and the product with comparative advantage in that country in the previous period, which is represented by the distance matrix *D*_*cp*_; *X* is the vector of country-time and product-time dummy variables to control for changes in country or product characteristics over time; and ε is the perturbation term.

To further separate product upgrading and loss of advantage (28), we include an interaction with product distance to examine the effect of product distance on the gain or loss of comparative advantage. Thus, Model (2) is developed to test Hypothesis 2:


(2) $$\begin{array}{l} y_{c,p,t}\;=\;\beta_{0}\;+\;\beta_{1}y_{c,p,t\;-\;1}\;+\;\beta_{2}y_{c,p,t\;-\;1}\;*\;Distance_{c,p,t\;-\;1}\\ \;+\;\beta_{2}(1\;-\;y_{c,p,t\;-\;1})\;*\;Distance_{c,p,t\;-\;1}\;+\;\beta_{3}X\;+\;\varepsilon \end{array}$$

If product *p* in country *c* does not have a comparative advantage in period *t*−1, the product distance will affect product upgrading in period *t* through β_2_, and this index indicates the role of product distance in product upgrading. If product *p* in country *c* already has a comparative advantage in period *t*−1, the product distance will affect the comparative advantage of the product in period *t* through β_1_, and this index indicates the role of product distance in preventing the product from losing its comparative advantage.

### Descriptive Statistics of Sample Data

A total of 46 medical devices products (Appendix 2) were identified by matching the 56 HS 6-digit medical device products provided by Yilmaz and Bayrak (35) with the HS 6-digit products in the global bilateral trade from 1995 to 2019 in the CEPII-BACI database.

The data of these medical device products were further processed. First, the exports of medical device products from each country/region in the world in 2019 were sorted in descending order to identify a total of 49 countries/regions whose total exports accounted for 99% of the world's total (Appendix 1). Second, the time series of the above countries/regions from 1995 to 2019 were examined, revealing that the export data of Belgium from 1995 to 1998 were missing. Therefore, this study covers a period of 20 years from 1999 to 2019 by reducing the sample time series. To reduce the influence of temporary factor changes on the sample, we grouped the data every 5 years to obtain 4 periods of data according to Hausmann and Klinger (28).^1^ Descriptive statistics of each variable in the product space are shown in Table 1.

**Table 1:** Descriptive statistics of each variable.

**Variable**	**Description**	**Obs**	**Mean**	**Std. Dev.**	**Min**	**Max**
RCA	Comparative advantage	47,334	1.273229	3.798106	0	110.5997
M	Binary comparative advantage	47,334	0.2776017	0.4478205	0	1
DISTANCE	Product distance	47,334	0.7139533	0.1323965	0.2463988	0.995772
OG	Potential opportunity gains	47,334	−0.0224291	0.2386412	−1.754847	1.597928
PCI	Product complexity	47,334	0.0641917	0.9090287	−2.230307	2.992397
PROMIX_MAX	Maximum proximity	47,334	0.4036323	0.254947	−0.1532028	1.155142
PROMIX_AVE	Average proximity	47,334	0.0910649	0.1921174	−0.4266038	0.3655137
NUM_LINK	Number of product links	47,334	43.45135	1.69681	30	45
DIVERSITY0	Country diversification	45,080	12.73061	4.779994	1	26
UBILITY0	Product ubiquity	45,080	13.56087	4.272888	4	26

## Empirical Results

Table 2 reports the regression results of Model (1), Model (2), and the robustness test.

**Table 2:** OLS model estimates.

	**Model (1)**	**Model (2)**	**Robustness test**
	***M***	***M***	**RCA**
L5.M	0.529^***^	0.509^***^	
	(0.022)	(0.093)	
L5.RCA			0.770^***^
			(0.043)
L5.DISTANCE	−0.966^***^		−1.275^*^
	(0.078)		(0.677)
L5.MxDISTANCE		−0.949^***^	
		(0.117)	
L5.M_dxDISTANCE		−0.980^***^	
		(0.089)	
HS6+YEAR_FE	YES	YES	YES
COUNTRY+YEAR_FE	YES	YES	YES
*N*	36,064	36,064	36,064
*R* ^2^	0.4779921	0.4780005	0.7077702
*F*	1104.814	970.275	715.2861

* *p < 0.1,*

*** *p < 0.01*.

In Model (1), the regression coefficient of the distance between medical device products in the previous period is significantly negative at the 1% level. Each 1% increase in the distance decreases the probability of developing innovative products by 96.6%. As discussed in Hypothesis 1, increased product distance reduces the likelihood of new product development, so medical device product innovation choices are less likely to be jump-started. Considering that the explained variable is a binary dummy variable, we use RCA to replace M for the robustness test. *L*5.*DISTANCE* is significantly negative at the 10% level, indicating that our regression results do not depend on the definition of the explained variable.

Model (2) considers the degree of influence of distance on the export of innovative products and on the abandonment of the export of existing products with comparative advantages. The coefficient of the *L*5.*M*× *DISTANCE* interaction, which represents the degree of influence of distance on the loss of advantage of a product, is significantly negative at the 1% level. This interaction indicates that the greater the product distance is, the more likely the products with existing comparative advantages will lose their advantages and vice versa. The coefficient of L5.M_d × *DISTANCE* interaction, which represents the degree of influence of distance on the export of innovative products, is significantly negative at the 1% level. This degree of influence indicates that the probability of products gaining comparative advantages decreases as the product distance increases. In other words, products with a closer distance are more likely to be upgraded.

The above conclusions are basically consistent with those of previous empirical research on product space and comparative advantage (28).

We further consider the heterogeneity of medical device product innovation across countries with different income levels as mentioned in the Introduction. The sample countries/regions are divided into 31 high-income, 11 upper middle-income, and 7 lower middle-income countries/regions based on the 2020 World Bank national income classification. From the perspective of sample size, the number of high-income countries/regions is more than twice that of upper middle-income countries/regions and lower middle-income countries/regions. This number is in line with the notion that the medical device industry is concentrated in high-income areas.

As shown in Table 3, the estimated coefficients of the explanatory variable *M* are all significantly positive at the 1% level, with no significant difference across income levels. The estimated coefficients of the explanatory variable *DISTANCE* are significantly negative at 5%, which generally conforms to the hypothesis. The estimated absolute value first increases and then decreases with the decrease of the national income level. The effect of distance on medical device product innovation in upper middle-income countries is far greater than that in lower middle-income countries.

**Table 3:** Model estimates of the effect of product distance on comparative advantage by income level.

**Income level**	**(1)**	**(2)**	**(3)**	**(4)**
	**Basic Reg**	**High income**	**Upper middle income**	**Lower middle income**
L5.M	0.529^***^	0.544^***^	0.505^***^	0.522^***^
	(0.022)	(0.027)	(0.047)	(0.042)
L5.DISTANCE	−0.966^***^	−0.908^***^	−1.140^***^	−0.602^**^
	(0.078)	(0.090)	(0.204)	(0.178)
HS6+YEAR_FE	YES	YES	YES	YES
COUNTRY+YEAR_FE	YES	YES	YES	YES
*N*	36,064	22,816	8,096	5,152
*R* ^2^	0.4779921	0.494581	0.516447	0.4913027
*F*	1,104.814	1,162.517	113.1427	94.81124

** *p < 0.05,*

*** *p < 0.01*.

Finally, we tested heterogeneity by region. The sample countries/regions are divided into 1 in Africa, 6 in America, 16 in Asia, 24 in Europe, and 2 in the Pacific region based on the United Nations Statistics Division (USUD) classification. Given that the sample size of Africa and the Pacific region is not large enough to run the fixed-effects model, we only report the regression results for America, Asia, and Europe (Table 4). The regression results show that the regression coefficients of *L*4.*DISTANCE* in the three regions are all significantly negative at the 1% level. Among them, medical device product innovation in Asia is most affected by product distance, losing 1.051 units of innovation opportunities for every additional unit of distance. America is relatively less affected by product distance, losing 0.883 units of innovation opportunities for every additional unit of distance. Compared with America, medical device innovation in Asia is more inclined to promote product diversification by selecting products with similar attributes. We will discuss the evolution path and product innovation choices of the medical device industry in major Asian countries in detail in Section Product Space Analysis of Medical Devices in Asia.

**Table 4:** Model estimates of the effect of product distance on comparative advantage by region.

**Region**	**(1)**	**(2)**	**(3)**	**(4)**
	**Basic Reg**	**America**	**Asia**	**Europe**
L5.M	0.529^***^	0.583^***^	0.498^***^	0.519^***^
	(0.022)	(0.040)	(0.047)	(0.030)
L5.DISTANCE	−0.966^***^	−0.883^***^	−1.051^***^	−1.009^***^
	(0.078)	(0.183)	(0.208)	(0.100)
HS6+YEAR_FE	YES	YES	YES	YES
COUNTRY+YEAR_FE	YES	YES	YES	YES
*N*	36,064	4,416	11,776	17,664
*R* ^2^	0.4779921	0.6279242	0.4707612	0.4886709
*F*	1104.814	1322.659	138.2341	1271.65

*** *p < 0.01*.

## Product Space Analysis of Medical Devices in Asia

As discussed above, Asia is one of the most dynamic markets for the development of the medical device industry. In the empirical analysis, the regression coefficient of product distance for Asia suggests the product innovation path in Asia. To explore the network characteristics of medical device product innovation in Asia, we construct a product space evolution diagram for medical device exports from major Asian countries. The map is based on the global medical device product space. Based on the sample processing in Sections Data Sources and Research Methods and Empirical Results, we also construct the product space network map of 1999, 2004, 2009, 2014, and 2019 with an interval of 5 years, and mark the products with obvious comparative advantages in each country on the nodes for analysis.

**Figure 1:**
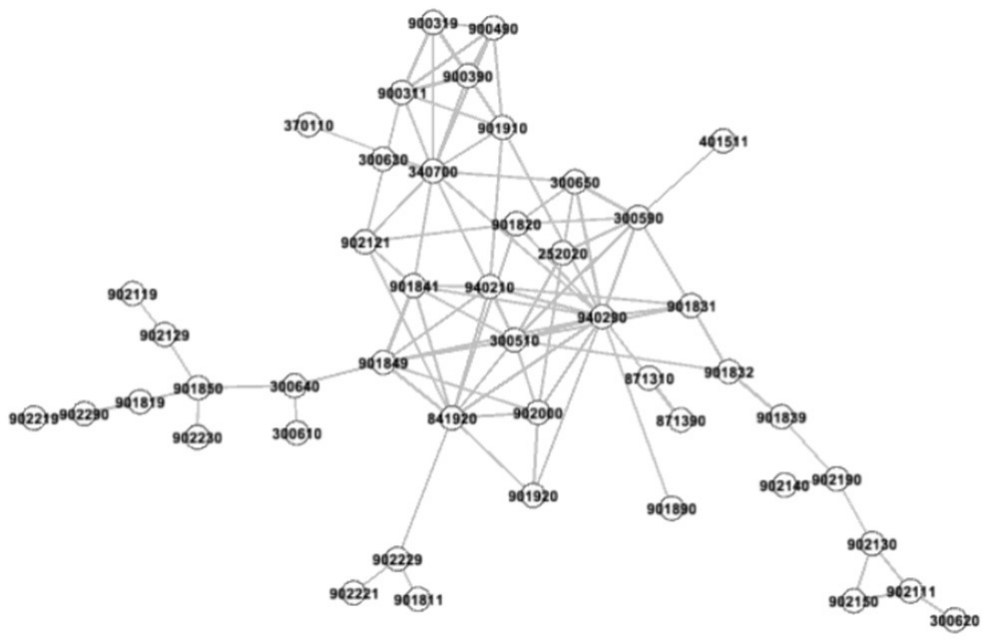
Medical device product space.

This article follows the product space network strategy of Hidalgo et al. (10). First, we calculate the maximum spanning tree of the average medical device product proximity matrix, establish 46 product nodes and 45 links of the product proximity network, and maximize the total proximity of the network. Second, we add further links, and select an appropriate proximity threshold to make the average degree of the network equal to 4. This is a known common rule for effective network visualization (33). Here, we choose to include all adjacent links ≥0.4 to form a network consisting of 46 nodes and 92 links. Third and finally, for better visualization, we use the ForceAtlas2 algorithm in Gephi to optimize the network view, add attributes to the size and color of the nodes, and adjust the layout to obtain a medical device product space map (Figure 1).

### Product Space Evolution

**Figure 2:**
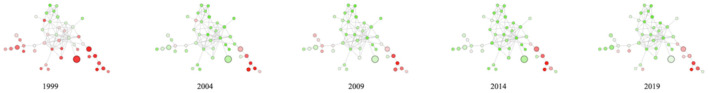
Evolution of medical device product complexity from 1999 to 2019.

Figure 2 shows the evolution of the complexity of medical device products from 1999 to 2019. The color of the label represents the PCI. The gradual change from green to red represents changes from low to high product complexity. Overall, the green nodes gradually spread to the edge over time, which means that the product complexity decreases along the dense area to the edge. Over time, an increasing number of countries have possessed the necessary factors of medical device products in the dense area. Among the two branches, the product complexity of the left branch decreases year by year, and the right branch remains highly complex.

**Figure 3:**
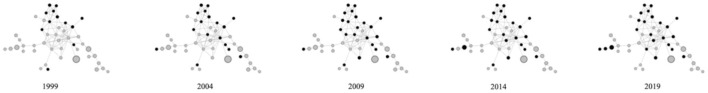
Evolution of medical device product space in China.

**Figure 4:**
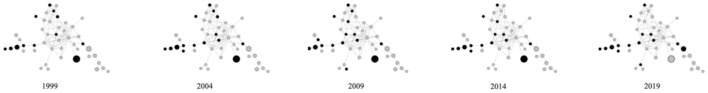
Evolution of medical device product space in Japan.

**Figure 5:**
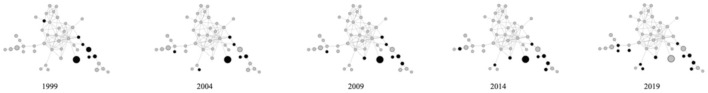
Evolution of medical device product space in Singapore.

**Figure 6:**
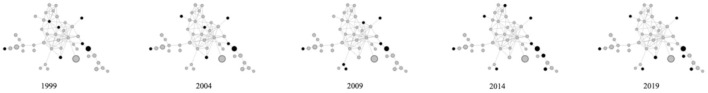
Evolution of medical device product space in Malaysia.

**Figure 7:**
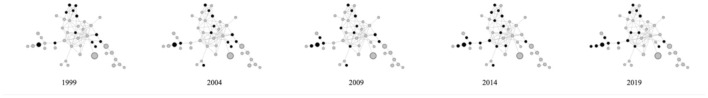
Evolution of medical device product space in South Korea.

We select the top five countries^2^ in Asia in terms of export value in 2019 as representative examples to analyze the dynamic evolution of product space in Asia. In the product space map, the node size represents the total world exports of the product in that year; black nodes represent products with obvious comparative advantages in representative Asian countries in each period; and gray nodes represent other products that do not have obvious comparative advantages (Figures 3–7).

In general, the product space maps of major medical device exporting countries in Asia have the following characteristics: (1) the number of medical device products with comparative advantages in the country is increasing year by year; (2) innovative medical device products tend to be created near existing products along the links; and (3) products with comparative advantages tend to cluster from the edge to the dense area.

As shown in Figures 2, 3, China exports a wide variety of medical device products with a low technical level. Its product space evolution has obvious overall characteristics. New products are mainly concentrated in the densely connected area, with significant growth from the edge to the center. However, this trend is similar to the evolution of PCI. Most medical device products with comparative advantages in China are still of low complexity.

As shown in Figure 4, Japan had the ability to produce complex products in the early evolution of medical device products (left branch). It evolved from the edge to the center along the product links, following the product space law. By also considering the evolution of PCI, it is found that Japan is gradually losing its advantage in complex products. The total number of medical device products with comparative advantages in Japan did not change from 2014 to 2019. The obvious feature is that Japan lost the comparative advantage of the largest node (HS 901890) in the product space map, but it gained a new comparative advantage in the complex product area in the lower right corner (HS 902190).

The total number of medical device products with comparative advantages in exports is not large for Singapore. It mainly produces and exports highly complex medical device products. Figures 2, 5 show that Singapore exported highly complex products in its early evolution, similar to Japan. From the perspective of product space, the two counties occupied the left and right complex areas, forming their own distinct but nonconflicting advantages. As the evolution progressed, Singapore not only always had the knowledge of complex products to maintain its advantage (right branch), but also made correct judgments about the frontier of product innovation to gain comparative advantages (HS 902230, HS 300610, and HS 901811).

Malaysia is the country with the lowest number of medical device products with comparative advantages and the fewest changes among the representative countries in Asia. The characteristic of Malaysia's product space evolution is that it always had a comparative advantage in some complex products (right branch) and successfully gained a comparative advantage in derivative products after 2014. However, other advantage products were always located at the edges of the product space and did not develop product diversity along the links.

Korea had relatively scattered products with comparative advantage in the initial stage, with an overall evolution trend of increasing advantage products from the edge to the dense area. Its evolution path in the left branch is similar to that of Japan. However, as shown in the product space map in 2019, South Korea lost some of its advantage products in the dense area (HS 340700 and HS 901820) and did not achieve breakthroughs in complex products.

The above facts suggest that (1) the evolution of medical device product space in Asian countries mostly has the characteristic of evolving along the maximum proximity between products; (2) there are obvious differences in the innovation strategies of medical device products between countries with different resource endowments; and (3) the reduction of product complexity is accompanied by technology diffusion, and more countries will have the opportunity to gain comparative advantages, while countries that have core knowledge may lose the advantage.

### Potential Product Distance Matrix Analysis

**Figure 8:**
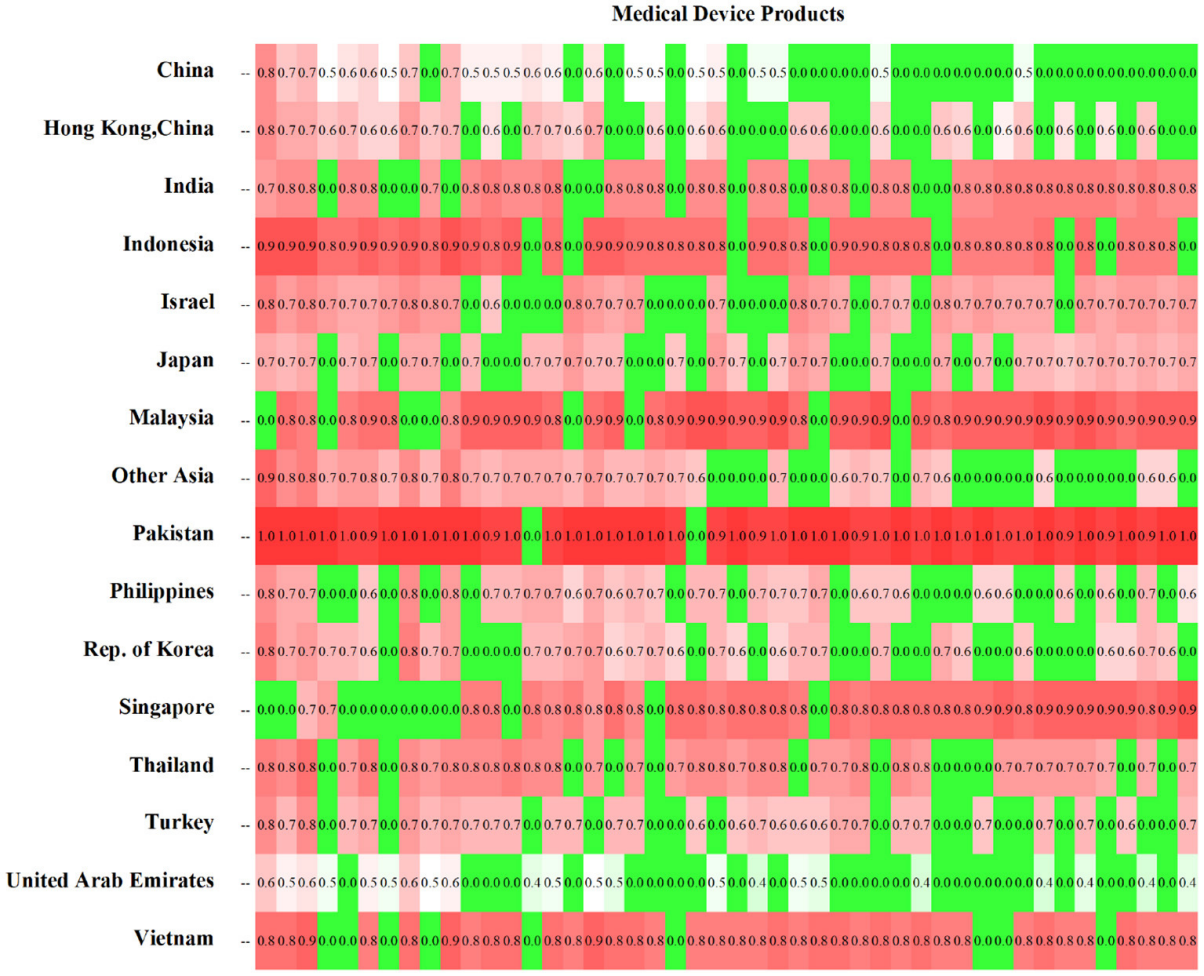
Potential product distance matrix by country/region.

The potential distance matrix of medical device products is a matrix composed of countries and medical device products. Due to space limitations, this article only discusses the potential product distance matrix of Asian countries/regions in 2019 as an example. The rows of the matrix are arranged from left to right in descending order of product complexity in that year. The cells are filled with potential product distances. As the distance increases, the color changes from green to red, as shown in Figure 8.

The potential product distance matrix provides the following information: (1) products currently produced by each country/region that already have a comparative advantage (dark green); (2) potential products that are likely to be produced by each country/region (light color); and (3) products that are highly unlikely to be produced by each country (dark red).

The data show that Saudi Arabia, China, and Hong Kong, China are the top three countries/regions in Asia in terms of product diversity with 24, 23, and 18 export products, respectively. They have a small overlap in high-complexity products and a large overlap in low-complexity ones. In terms of the number of light-colored cells, Saudi Arabia is the most capable country in Asia to produce diversified medical device products (38 products), followed by China (27) and Hong Kong, China (21). A dark-colored cell means that the country does not have the capacity to produce the product, so it is highly unlikely to be produced by that country. Pakistan, Malaysia, Indonesia, and Vietnam will still be at a disadvantage in the medical device industry in the future.

### Potential Product Gains

**Figure 9:**
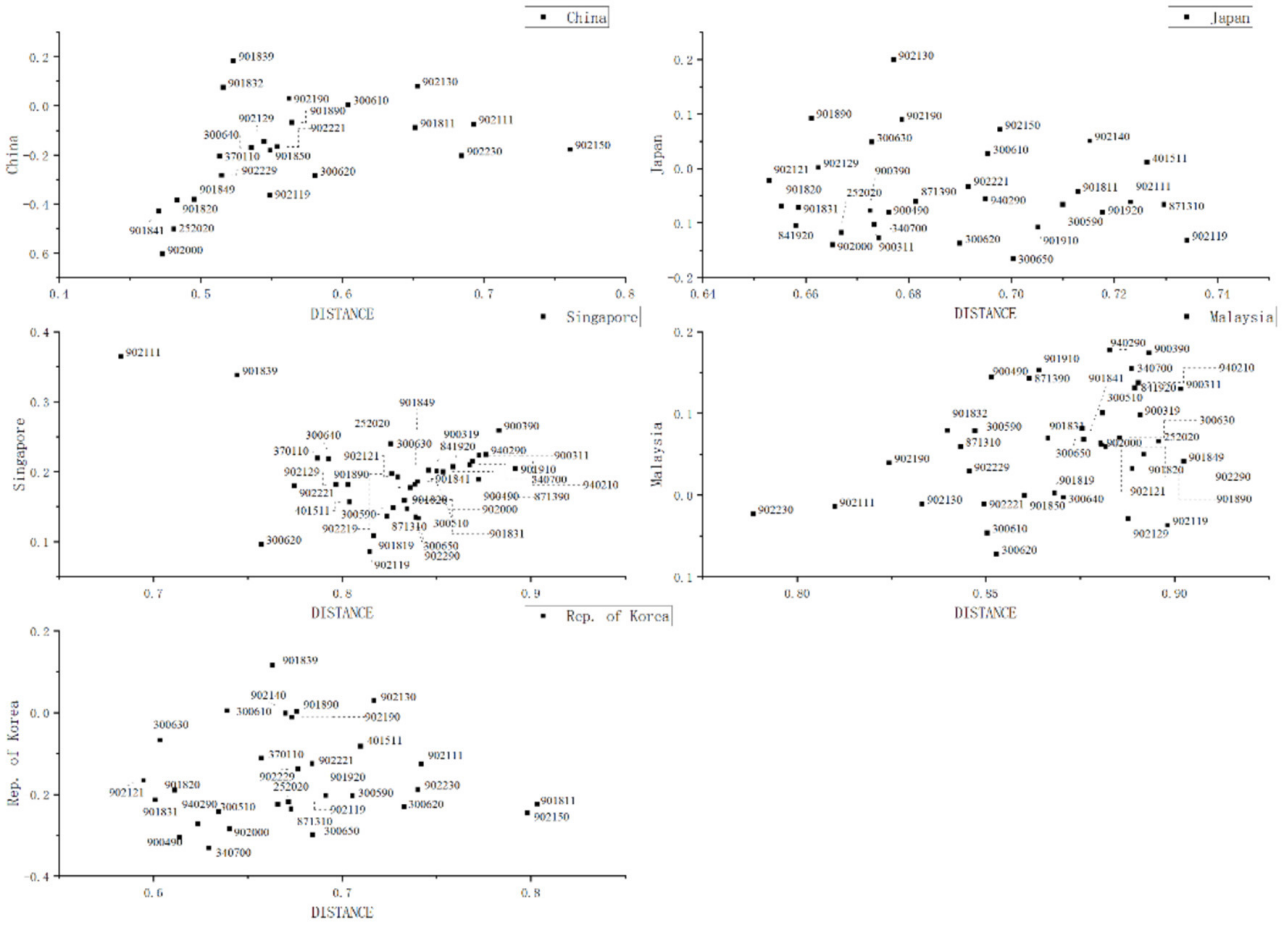
Distance-opportunity gain graphs for potential medical device products.

We continue to analyze the potential product gains for these representative countries with China, Japan, Singapore, Malaysia, and South Korea as examples. The product space evolution analysis reveals the shortcomings and suggestions for of the current medical device industry of each country to a certain extent, such as how China can overcome the lack of high-complexity products, whether Malaysia should seek product diversity, how Japan and South Korea can recover the advantage in complexity, how Singapore continues to identify high-complexity products and how India prevented the loss of comparative advantage. This section will create distance-opportunity gain graphs for potential medical device products (Figure 9) and provide suggestions for these countries to further pursue new opportunities for product innovation.

The distance-opportunity gain graph for China is consistent with its evolution of product space. It can be seen that opportunity gains are negative for most products in China. The negative sign comes from PCI. This measurement means that the gains of these products are below average. China can consider two steps in its pursuit of high-tech products: First, it can continue to upgrade along the shortest product distance along the original path in the short term. These products are still of low complexity and provide no significant potential opportunity gains but are relatively easy to implement. Examples are HS 252020, HS 901820, HS 901841, HS 901849, and HS 902000. Second, it should explore products that have both positive gains and the shortest distance in the medium and long term, such as HS 901832 and HS 901839.

Japan and South Korea can choose to seek short-term breakthroughs along the shortest path. An example is HS 902121. On the other hand, due to their relatively rich technical knowledge base of medical device products, their reachability distance for seeking high-tech products is relatively short. In other words, it is easier for them to realize the jump to complex products. Hence, the key is to identify high-potential products. Therefore, we suggest that the two countries prioritize the development of high-gain products at an appropriate distance. For example, Japan can consider HS 902130, which is at an appropriate distance of 0.677 and has the second highest complexity, HS 901890, which is at a shorter distance and still provides above-average gains, and HS 902190, which is at a slightly farther distance and has the fifth highest complexity. For Korea, the product we identify is HS 300610, which can reach the highest-complexity product in the shortest distance (ranked 7th in complexity in 2019) and provides above-average potential opportunity gains.

Singapore is the country in Asia that has the production capacity for complex products. As shown in the opportunity gain graph, HS 902111, which is at a distance of 0.6826 and ranked third in complexity, and HS 901839, which is at a distance of 0.744 and ranked fourth in complexity, are relatively discrete, are at a short potential distance, and provide high potential opportunity gains. The other products appear to be concentrated at a longer distance and provide smaller opportunity gains; in other words, they are in an area that is unlikely to be developed in the future.

In the product space evolution map, we found that Malaysia has mastered some high-tech products. However, there is a lack of product diversity, which is explained by the potential distance-gain graph. We found that all medical device products are at a distance of >0.7 in Malaysia, which is very unfavorable for product derivative innovations. From the perspective of opportunity gains, HS 902230 should be prioritized for breakthrough innovation.

## Conclusions And Directions For Further Research

### Conclusions and Implications

This article answers the question of how to evaluate the feasibility of medical device innovation and production in a specific environment from the perspective of national output and capability. To evaluate this innovation, we consider the systematic requirements for the innovative development of medical devices and employ the product space approach at the forefront of complexity economics. Furthermore, taking the medical device product space in Asia as an example, we evaluate the innovation potential and opportunities of potential medical device products in representative countries, supplement the analysis of current national policies on medical device products with export advantages and potential export advantages for national policy-makers and stakeholders, and provide guidance for formulating policies in line with national industrial development. The following conclusions are drawn:

First, empirical analysis confirms that a close product distance improves the feasibility of the development of a new medical device. Further analysis by separating comparative advantages suggests that a close product distance helps maintain the existing comparative advantages and promotes product innovation by gaining potential comparative advantages to enhance the diversity of medical device products in a country.

Second, the product space evolution map of medical devices in Asia reveals that medical device product innovation and industrial evolution in major Asian countries mostly evolve along the minimum product distance but show heterogeneity in the selection of evolution paths. By also considering the evolution of product complexity, it is found that the reduction of product complexity is accompanied by technology diffusion, and more countries will have the opportunity to gain comparative advantages. For example, the diversity of low-complexity products is a specific characteristic of China.

Third, the potential product distance matrix visualizes the diversity of medical device exports in Asia. Among Saudi Arabia, China, and Hong Kong, China has the most diverse medical device products, while Pakistan, Malaysia, Indonesia, and Vietnam lack diversity. The potential product gain analysis provides-policymakers and stakeholders with a perspective focused on medical device innovation. We recommend prioritizing the development of high-gain products at the shortest distance. For example, for China, although there is a wide variety of medical device products, they are concentrated in low-complexity areas. Based on the shortest distance and the greatest potential gain, we identify the complex product HS 901832 (Medical, surgical instruments and appliances; tubular metal needles and needles for sutures) with the greatest potential for breakthroughs.

These conclusions can help countries identify the necessary capabilities that they already have for the development of medical device products and determine next steps. The conclusions are not limited to Asia but can be extended to all regions where statistics are available.

### Limitations and Directions for Further Research

This study has two limitations. The first lies in the scope of medical device products. There is no uniform classification for medical device products, and fewer products are eventually included in trade studies. Therefore, our conclusions depend to a certain extent on the product list we selected. In the future, we will continue to focus on the list of medical device products in this field of research. The second limitation is the partial product space. We only considered a list of 46 manufactured medical device products. This product space may ignore the impact of other related industries on the medical device industry. In the future, we will build a complete product space on the basis of improving the list of medical device products and comprehensively consider the impact of related industries.

## Data Availability Statement

The original contributions presented in the study are included in the article/Supplementary Material, further inquiries can be directed to the corresponding author/s.

## Author Contributions

All authors undertook research, writing, and review tasks throughout this study and have read and agreed to the published version of the manuscript.

## Funding

This work was supported by the Major Program of the National Social Science Foundation of China [Grant Number 20&ZD124], the National Social Science Foundation of China [Grant Number 21CJY024, 20BJL040, 19BJL108, 19BJL185, and 20BJY110], the National Natural Science Foundation of China [Grant Numbers 71773115, 72174180, 72074195, 71973129, 72072162, 72173073, 71503003, and 72174112], the Philosophy and Social Science Program of Zhejiang [Grant Numbers 22NDQN290YB, 22QNYC13ZD, and 21NDYD097Z], and the Humanity and Social Science Foundation of Ministry of Education of China [Grant Numbers 21YJA790043, 21YJA630037, 19YJA790107, 19YJA630092, 18YJA790088, and 21YJCZH213].

## Conflict of Interest

The authors declare that the research was conducted in the absence of any commercial or financial relationships that could be construed as a potential conflict of interest.

## Publisher's Note

All claims expressed in this article are solely those of the authors and do not necessarily represent those of their affiliated organizations, or those of the publisher, the editors and the reviewers. Any product that may be evaluated in this article, or claim that may be made by its manufacturer, is not guaranteed or endorsed by the publisher.

## Footnotes

^1^We tested the optimal lag order of the model before the empirical test. The BIC and HQIC information criteria indicated an optimal lag order of 4 and 5, respectively, which was basically consistent with the five-year panel study by Hausmann and Klinger (28).

^2^The top five countries in terms of medical device exports in Asia in 2019 are China (41%), Japan (14%), Singapore (12%), Malaysia (7%), and South Korea (6%).
