# Supplementary material for: Medical Device Product Innovation Choices in Asia: An Empirical Analysis Based on Product Space
Source: Front Public Health. 2022 Apr 13;10:871575. doi: 10.3389/fpubh.2022.871575 (PMC9043244; doi:10.3389/fpubh.2022.871575)
Supplement: Supplementary file 1 [file Data_Sheet_1.pdf]

Appendix

I. 49 countries/regions whose total exports accounted for 99% of the world's total

Code	Country	Continent	Export 2019 in thousands current USD	Proportion	Accounted Proportion
**842**	USA	America	50214719.26	18.5381225	18.5381225
**276**	Germany	Europe	30496781.67	11.2587123	29.7968348
**156**	China	Asia	24325735.16	8.98050344	38.7773382
**528**	Netherlands	Europe	20567069.91	7.59289044	46.3702287
**484**	Mexico	America	16488414.63	6.08714447	52.4573731
**372**	Ireland	Europe	14760936.56	5.44939919	57.9067723
**757**	Switzerland	Europe	12303616.18	4.54221287	62.4489852
**56**	Belgium	Europe	9915911.724	3.66072716	66.1097124
**251**	France	Europe	8633417.702	3.18725978	69.2969721
**392**	Japan	Asia	8358508.596	3.08576965	72.3827418
**826**	United Kingdom	Europe	7215220.855	2.66369404	75.0464358
**702**	Singapore	Asia	6988082.756	2.57983986	77.6262757
**381**	Italy	Europe	6140703.084	2.26700672	79.8932824
**458**	Malaysia	Asia	4138534.873	1.52785215	81.4211346
**188**	Costa Rica	America	3890864.228	1.43641782	82.8575524
**410**	Rep. of Korea	Asia	3317555.32	1.22476533	84.0823177
**616**	Poland	Europe	3258378.746	1.20291869	85.2852364
**40**	Austria	Europe	2946336.94	1.08772002	86.3729564
**376**	Israel	Asia	2636650.548	0.9733909	87.3463473
**124**	Canada	America	2310952.351	0.8531506	88.1994979
**208**	Denmark	Europe	2265067.877	0.8362111	89.035709
**36**	Australia	Pacific	1977361.865	0.72999664	89.7657057
**490**	Other Asia	Asia	1954693.424	0.72162798	90.4873336
**724**	Spain	Europe	1904869.24	0.70323403	91.1905677
**246**	Finland	Europe	1873921.529	0.69180884	91.8823765
**752**	Sweden	Europe	1752070.292	0.64682416	92.5292007
**214**	Dominican Rep.	America	1725854.606	0.63714593	93.1663466
**704**	Viet Nam	Asia	1715465.441	0.63331049	93.7996571
**764**	Thailand	Asia	1637525.387	0.60453681	94.4041939
**203**	Czechia	Europe	1620174.805	0.59813137	95.0023253
**699**	India	Asia	1612395.36	0.59525938	95.5975847
**348**	Hungary	Europe	1315865.164	0.48578724	96.0833719
**344**	Hong Kong, China	Asia	1278337.846	0.47193301	96.5553049
**792**	Turkey	Asia	814698.524	0.30076801	96.8560729
**76**	Brazil	America	522276.723	0.19281259	97.0488855
**554**	New Zealand	Pacific	511965.198	0.18900581	97.2378913
**579**	Norway	Europe	511255.379	0.18874376	97.4266351

**440**	Lithuania	Europe	473514.995	0.17481087	97.601446
**608**	Philippines	Asia	468875.575	0.17309811	97.7745441
**784**	United Arab Emirates	Asia	464472.638	0.17147264	97.9460167
**586**	Pakistan	Asia	451850.403	0.1668128	98.1128295
**705**	Slovenia	Europe	400572.485	0.14788217	98.2607117
**360**	Indonesia	Asia	331268.826	0.12229685	98.3830085
**620**	Portugal	Europe	319732.692	0.11803798	98.5010465
**642**	Romania	Europe	310948.706	0.11479513	98.6158416
**100**	Bulgaria	Europe	286006.351	0.10558698	98.7214286
**703**	Slovakia	Europe	275422.2	0.10167956	98.8231082
**788**	Tunisia	Africa	268856.636	0.0992557	98.9223639
**643**	Russian Federation	Europe	245233.939	0.09053475	99.0128986

II. Medical devices List

CODE	Sector	Definition
**252020**	Minerals	Plasters; (consisting of calcined gypsum or calcium sulphate), whether or not coloured, with or without small quantities of accelerators or retarders
**300510**	Chemicals	Dressings, adhesive; and other articles having an adhesive layer, packed for retail sale for medical, surgical, dental or veterinary purposes
**300590**	Chemicals	Wadding, gauze, bandages and similar articles; (excluding adhesive dressings), impregnated or coated with pharmaceutical substances, packaged for retail sale
**300610**	Chemicals	Pharmaceutical goods; sterile surgical catgut, suture materials, tissue adhesives, laminaria, laminaria tents and absorbable surgical or dental haemostatics
**300620**	Chemicals	Pharmaceutical goods; blood-grouping reagents
**300630**	Chemicals	Pharmaceutical goods; opacifying preparations for x-ray examinations, diagnostic reagents designed to be administered to the patient
**300640**	Chemicals	Pharmaceutical goods; dental cements and other dental fillings, bone reconstruction cements
**300650**	Chemicals	Pharmaceutical goods; first aid boxes and kits
**340700**	Chemicals	Modelling pastes; preparations known as dental wax, put up in sets, in packings for retail sale or in plates, horseshoe shapes, sticks or similar forms, other
**370110**	Chemicals	Photographic plates and film; for x-ray, in the flat, sensitised, unexposed, of any material other than paper, paperboard or textiles
**401511**	Chemicals	Rubber; vulcanised (other than hard rubber), surgical gloves
**841920**	Machinery	Sterilizers; for medical, surgical or laboratory use, not used for domestic purposes
**871310**	Vehicles	Carriages for disabled persons; not mechanically propelled
**871390**	Vehicles	Carriages for disabled persons; mechanically propelled
**900311**	Machinery	Frames and mountings; for spectacles, goggles or the like, of plastics
**900319**	Machinery	Frames and mountings; for spectacles, goggles or the like, of materials other than plastics
**900390**	Machinery	Frames and mountings; parts for spectacles, goggles or the like
**900490**	Machinery	Spectacles, goggles and the like; (other than sunglasses) corrective, protective or other
**901811**	Machinery	Medical, surgical instruments and appliances; electro-cardiographs
**901819**	Machinery	Medical, surgical instruments and appliances; electro-diagnostic apparatus (including apparatus for functional exploratory examination or for checking physiol
**901820**	Machinery	Medical, surgical instruments and appliances; ultra-violet or infra-red ray apparatus
**901831**	Machinery	Medical, surgical instruments and appliances; syringes, with or without needles
**901832**	Machinery	Medical, surgical instruments and appliances; tubular metal needles and needles for sutures
**901839**	Machinery	Medical, surgical instruments and appliances; catheters, cannulae and the like
**901841**	Machinery	Dental instruments and appliances; dental drill engines, whether or not combined on a single base with other dental equipment

**901849**	Machinery	Dental instruments and appliances; other than dental drill engines
**901850**	Machinery	Ophthalmic instruments and appliances
**901890**	Machinery	Medical, surgical or dental instruments and appliances; n.e.s. in heading no. 9018
**901910**	Machinery	Mechano-therapy appliances; massage apparatus and psychological aptitude-testing apparatus
**901920**	Machinery	Therapeutic respiration apparatus; ozone, oxygen, aerosol therapy apparatus; artificial respiration or other therapeutic respiration apparatus
**902000**	Machinery	Breathing appliances and gas masks; excluding protective masks having neither mechanical parts nor replaceable filters and excluding apparatus of item no. 901
**902111**	Machinery	Orthopaedic or fracture appliances; artificial joints
**902119**	Machinery	Orthopaedic or fracture appliances; crutches, surgical belts and trusses, splints and other fracture appliances
**902121**	Machinery	Dental fittings; artificial teeth
**902129**	Machinery	Dental fittings; other than artificial teeth
**902130**	Machinery	Artificial parts of the body
**902140**	Machinery	Hearing aids (excluding parts and accessories)
**902150**	Machinery	Pacemakers; for stimulating heart muscles (excluding parts and accessories)
**902190**	Machinery	Appliances; worn, carried or implanted in the body, to compensate for a defect or disability
**902219**	Machinery	Apparatus based on the use of x-rays, including radiography or radiotherapy apparatus; for other than medical, surgical, dental or veterinary uses
**902221**	Machinery	Apparatus based on the use of alpha, beta or gamma radiations, including radiography or radiotherapy apparatus; for medical, surgical, dental or veterinary us
**902229**	Machinery	Apparatus based on the use of alpha, beta or gamma radiations, including radiography or radiotherapy apparatus; (for other than medical, surgical, dental or v
**902230**	Machinery	X-ray tubes
**902290**	Machinery	Apparatus based on use of x-rays and similar; parts and accessories (x-ray generators, tubes, high tension generators, control panels and desks, screens, exam
**940210**	Textiles	Chairs; dentists', barbers' or similar chairs having rotating as well as both reclining and elevating movements, and parts thereof
**940290**	Textiles	Furniture; for medical, surgical, veterinary use (eg operating tables, examination tables, hospital beds with mechanical fittings) and parts thereof
